# New route for hollow materials

**DOI:** 10.1038/srep32107

**Published:** 2016-08-24

**Authors:** C. M. Rivaldo-Gómez, F. F. Ferreira, G. T. Landi, J. A. Souza

**Affiliations:** 1 Centro de Ciências Naturais e Humanas, Universidade Federal do ABC, Santo André – SP, 09210-580, Brazil

## Abstract

Hollow micro/nano structures form an important family of functional materials. We have used the thermal oxidation process combined with the passage of electric current during a structural phase transition to disclose a colossal mass diffusion transfer of Ti ions. This combination points to a new route for fabrication of hollow materials. A structural phase transition at high temperature prepares the stage by giving mobility to Ti ions and releasing vacancies to the system. The electric current then drives an inward delocalization of vacancies, condensing into voids, and finally turning into a big hollow. This strong physical phenomenon leading to a colossal mass transfer through ionic diffusion is suggested to be driven by a combination of phase transition and electrical current followed by chemical reaction. We show this phenomenon for Ti leading to TiO_2_ microtube formation, but we believe that it can be used to other metals undergoing structural phase transition at high temperatures.

Hollow structures with high surface-to-volume ratios and loading capacity have attracted great attention as an important group of functional materials^1^. The diverse set of properties paves the way to the design of materials with multifunctional architectures, suited for a broad range of applications^2^,^3^,^4^. Indeed, the fabrication of micro-nanostructures has been one of the aims that guide the basic research in condensed matter physics. As far as this point is concerned, there is a considerable interest in methodologies to fabricate and, consequently, use the physical and chemical properties of hollow micro-nanostructures. Controlled synthesis process would allow the formation of micro-nanostructures with multifunctional architecture in which several applications can be anticipated^5^,^6^,^7^. One particularly important class of micro-nanostructure materials is that of transition metal oxides, which are good candidates for various applications in different fields such as nanofluidic, drug vectorization^8^, catalysis, energy storage and conversion^9^, spintronic devices, and biomedicine^10^,^11^, or as metal-oxide electrodes in lithium-ion batteries and super-capacitors^12^.

Over the last few years, great efforts have been done in order to obtain hollow (tubes and spheres) structures of several materials. The most well-known hollow materials are carbon nanotubes and fullerene, whose underlying mechanism comes simply from self-organization^13^,^14^. Besides self-assembly, hollow structures can be produced by coating^15^, using sacrificial templates^16^, sol-gel method^17^ or by simply rolling up layered materials. In most cases, a regular geometry and the presence of blockages in the obtained hollow structures configure an extra technological difficulty. More recently, there has been an important breakthrough based on the Kirkendall effect^18^, originally studied in the field of metallurgy, where the formation of hollow spherical nanocrystals was observed^19^. This effect is based on the fact that outward diffusion of metal atoms from the core is faster than inward diffusion of reactive species, so that vacancy movement takes place resulting in void formation. After this seminal work, scientists have constructively applied this effect for synthesizing hollow nanostructures^20^,^21^,^22^.

However, when trying to extend these results to micro-structures, one finds that the mass transport due to the Kirkendall effect is not sufficient to produce a hollow material. Alternative mass transport mechanisms are therefore necessary. In this paper, we present a method to produce hollow metal-oxide micro-tubes starting from metallic micro-wires. This is accomplished using a thermal oxidation process combined with the passage of electric current, as the system passes through a structural phase transition. Our results disclose a power physical phenomenon and open the way to a new route to fabricate hollow micro-structures. We believe that, first, the thermal oxidation along with the phase transition prepare the stage by creating a large number of vacancies into the system. The electric current then drives the delocalization of vacancies, which condense into voids, and finally turn into a hollow. This strong physical phenomenon, leading to a colossal mass transfer through ionic diffusion, is therefore suggested to be driven by a combination of phase transition and electrical current followed by chemical reaction.

By carrying out *in situ* electrical resistivity measurements we are able to assist simultaneously the oxidation process during the passage of electrical current. As we shall see, this combination along with the occurrence of a structural phase transition reveals a colossal diffusion phenomenon. However, first, we show the oxidation process and core-shell formation on metallic microwires when only temperature and annealing time are involved. The oxidative chemical reaction involves a process where a thin oxide layer is formed on the metal surface, followed by simultaneous outward diffusion of metal ions through the oxide scale and inward diffusion of oxygen into the core. This is observed for our heat-treated sample without the passage of an electrical current. After the thermal oxidation procedure took place, all samples showed a white color, typical of titanium dioxide. Figure 1(a–c) display SEM images of metallic Ti microwires heat treated in air and annealed for different times *t* = 0, 1, and 2 h at T = 1050 °C. The first microwire, Fig. 1(a), shows a well-defined core-shell type structure – the metallic Ti core and a titanium oxide shell.

The X-ray diffraction measurements, with Rietveld refinements^23^,^24^, of the microwire, shown in Fig. 1(a), (see Fig. S1 in Supplemental Material), indicated the presence of two different crystallographic phases: TiO_2_ (91%) and Ti_2_O (9%) with *P*4_2_*/mnm* and *P*6_3_*/mmc* space group symmetries, respectively. The second microwire oxidized for 1 h, Fig. 1(b), shows an outer layer with a different morphology. This outer ring-like layer is denser and XRD data indicate TiO_2_ phase. The inner layer is more granular and also belongs to the TiO_2_ crystal phase, but with a deficiency in oxygen content. The third microwire was exposed to a longer annealing time, *t* = 2 h, at T = 1050 °C, which turned out to produce a completely oxidized core covered with a denser thin layer. The external diameter of the core/shell microwires is 154, 173, and 201 μm for *t* = 0, 1, 2 h, respectively. Despite the possible migration of Ti ions^25^, it is clearly seen in the images that there is no significant formation of voids at the Ti/TiO2 interface, which could reveal the presence of the Kirkendall effect. Here, we believe that the Kirkendall effect is not sufficient to generate hollows in micrometer scale due to the absence of high mobility of cations and a driven force to coalesce the voids.

Conversely, in Fig. 1(d–f) we present the same experiment, but in the presence of a direct electric current applied along the wire. No annealing time was employed in this case, just the upward and downward temperature ramps. Figure 1(d) was obtained using 10mA and Fig. 1(e) with 0.1 mA. Figure 1(f) shows several segments of long Ti oxide microtubes obtained during different runs. As one can observe, the application of an electric current leads to the formation of micro-tubes of remarkably homogeneous shape. The external and internal diameters of the micro-tubes were in the range of 240 and 140 μm respectively which, we note, is larger than the original diameter of the wire, which was 127 μm. We also point out that the experiment is fast, clean, and easily reproducible. X-ray diffraction reveals microtubes with TiO_2_ rutile single phase and others with TiO_2_ rutile major phase coexisting with traces of hexagonal Ti metallic (see Fig. 1S in Supplemental Materials). The surface morphology of the obtained microtubes is very interesting. The microstructure of the surface of the microtube shown in Fig. 1(d) is displayed in the Supplemental Materials, Figs 2S and 3S. One can see several flat planes forming regular polyhedron on the surface of the as obtained tube. It is very dense and there is no pore. When the tube is annealed for a longer time at the highest temperature, the microstructure becomes more granular and some pores appear.

Figure 2 shows *in situ* electrical resistivity measurements with an applied current of 10 mA as the oxidation process takes place for a metallic titanium microwire. As the temperature increases from room temperature, the electrical resistivity also increases as is expected for any metal. However, at T = 850 °C, the electrical resistivity increases sharply four orders of magnitude, during a time span lasting no longer than 2 minutes, in an unexpected behavior. At first glance, it seems that the titanium metallic percolation path breaks down - the metallic medium is consumed and loses continuity, breaking down the path of electrical current. As temperature increases further, the electrical resistivity increases even more and reaches values compared to oxides.

On the other hand, there are sintering methods, spark plasma or field assisted sintering, which use a pulsed direct electrical current to perform high speed consolidation of powder samples^26^,^27^,^28^. It uses very high electrical current typically from 1 to 10 kA, which generates high localized temperatures, up to ten thousand Celsius. This way of heating allows the application of a very high heating rate which in turn promotes diffusion and enhances densification over grain growth. In our case, the Joule heating effect is very small. The magnitude of the used electrical current is very small, 1 to 10 mA, when compared to spark plasma sintering. We have estimated the temperature rise due to the Joule heating effect and found it to be negligible, of ~10 °C^29^.

We have also measured Raman spectroscopy. Those results were included in the Supplemental Materials. Figure 4S presents the unpolarized Raman spectrum of TiO_2_ microtube sample and Table 1S of the Supplemental Materials the vibrational bands assignment^30^. The first-order modes E_g_, A_1g_, and B_2g_ and the second order Raman scattering of rutile phase dominate the observed Raman spectra. Nonetheless, the E_g(1)_ band of anatase (around 1%) was detected as a very tiny band at 140 cm^−1^. Another interesting result is on the microstructure of the surface of the microtube. Following the reported redshift in the A_1g_ mode as function of crystallite size^31^,^32^, we found that the average crystallite size for the TiO_2_ microtubes is ~5 nm. At first glance, the morphology of the rutile microtubes appears to be a tubular nanomosaic one. However, more detailed studies need to be performed in order to clarify the long range morphology which will be published elsewhere. Furthermore, energy dispersive spectroscopy (EDS), see Fig. 5S in the Supplemental Materials, allows to map the distribution and relative proportion over the scanned area of the microtube. The results reveal that the mass composition of Ti and Oxygen changes only slightly depending on the region.

On cooling down, the electrical resistivity increases exponentially, which indicated a thermally activated behavior corroborating the semiconducting behavior of TiO2. The thermally activated behavior in semiconductors is described by the Arrhenius equation, ρ(T) = ρ_0_exp(−E_A_/kT), where ρ_0_ is a constant, E_A_ the activation energy, k is the Boltzmann constant, and T is the absolute temperature. At high temperatures, the activation energy is equal to half of the band gap energy (Eg)^33^. From the temperature dependence of the electrical resistivity, the activation energy is found to be E_A_ = 0.72 eV. The band gap energy of TiO_2_ rutile is 3.0 eV^34^. We suggest that this value is influenced by both the presence of interstitial Ti species due to fast migration in the crystal lattice and oxygen vacancies as structural defects.

Considering the drastic change in the morphology, one is induced to think that the microwire had completely oxidized in a regular and homogeneously form, but microtubes were found. Taking into account the area of the metal wire and the resulting area of the microtube along with difference in the molar volume of Ti and TiO_2_, one can conclude that there is mass conservation. So the metal neither evaporates nor dropped away, indicating a diffusion process followed by chemical reaction forming TiO_2_. The extraordinarily short time span during which the process occurs is worth noting. It points to a colossal mass transfer mechanism. A rough estimate of the mass flux, taking into account the surface area and a temperature interval from the jump in Fig. 2, would lead to a 1.5 × 10^10^ atoms/μm^2^·s.

It is very interesting and important for the following interpretation that the jump in the electrical resistivity takes place close to the structural phase transition of the Ti metal from a hexagonal close-packed crystal structure to a body-centered cubic crystal structure at 882 °C^35^. The inset of Fig. 2 shows ρ(T) measurements of a metallic Ti microwire in inert (argon flow) atmosphere up to T = 960 °C. At T = 882 °C, this structural phase transformation is reflected in a sharp, but small drop in the electrical resistivity. On cooling, the transition is rather broad and takes place at a lower temperature. We have observed that after the microwires subjected to this process with inert atmosphere do not result in microtubes by doing the thermal oxidation with electrical current as the virgin microwires do. Differential scanning calorimetry also reveals this structural phase transition as shown in the inset of Fig. 2.

In order to shed light on the nature of the mechanism responsible for the microtube formation, we have aborted the heating process depicted in Fig. 2 at certain specific temperatures and cooled down the sample quickly to room temperature. Figure 2 sketches the process by showing SEM images at different stage of the process. In the initial stage, we have a Ti metallic microwire with a diameter of 127 μm. The second stage, around T = 830 °C, just below the jump in the electrical resistivity, a TiO_2_ thin layer is formed at the surface of the microwire. This result reveals that just below the phase transition almost all the metal is still in the core. At T = 860 °C, it is observed a partially-oxidized hollow microwire, but still containing metal Ti inside. It is very interesting that there is two eccentric microtubes – an outer and larger TiO_2_ microtube and an inner partially oxidized metallic Ti microtube. It seems that the formation of voids at the center competes with those at the interface. Additional examples of microtube formation are shown in Fig. S6 of Supplemental Material. One can suggest that the internal metallic microtube is initially linked to the shell through multiple filaments. If the filaments break before the core is completely consumed (in this case induced by stopping the process), this internal microtube is left inside the outer microtube, as shown in Fig. 2. On the other hand, as we believe that the colossal mass transfer is a cascade-like effect, aborting at the middle of the process may bring about misleading interpretation of the process. In the last illustrated stage, at T = 900 °C, one finds a microtube with dimensions about 125 and 214 μm for the inner and outer diameter, respectively.

Figures 1(d–f) and 2 reveal a remarkable result where a huge hollow is produced when a small electrical current is applied simultaneously to the oxidation process during the occurrence of a structural phase transition. In order to have a better understanding of the phase transition evolution we have collected some XRD patterns as a function of temperature during the oxidation process. The most intense and important reflections are shown in Fig. 3(a). The complete patterns are shown in Fig. S7 of Supplemental Material. At T = 830 °C, there is only the presence of the α-Ti metallic phase. We have observed that the phase transition starts at T = 860 °C where the β-Ti metallic phase appears, coexisting with the α-Ti one as well as a TiO_2_ phase with bimodal unit cell parameters. As the temperature increases, the β-Ti phase gradually increases and then, surprisingly, changes back to the α-Ti phase, as shown in Fig. 3(a), where one can see the evolution of the most intense (011) Bragg peak of β-Ti as a function of temperature. The volume fractions of each phase are shown in Table S1 of the Supplemental Material. It is important to emphasize that the structural phase transition from α to β starts as expected, but during the oxidation it turns back, resulting in only α-Ti phase coexisting with the TiO_2_ at T = 980 °C. A small amount of oxygen shifts the structural transition to higher temperatures^35^.

The precise details of how the electric current and the structural phase transition combine to yield this mass transfer mechanism are not entirely understood. Notwithstanding, some possibilities may be elucidated. It is recognized that during the phase transition, diffusive movement of ions to create the new structure takes place. In this case, from hexagonal to cubic, there is the diffusion and dislocation of atoms lying on plane (011) of the former phase (orange spheres) to the plane (011) of the latter one (grey spheres) as illustrated in Fig. 3(b). One can see the atoms located at the middle of the α-Ti will move towards the corners of the β-Ti phase. Diffusion of substitutional lattice atoms involves a large number of defects creating vacancies. Delocalization of vacancies will bring about the formation of void within the low temperature phase. After vacancies condensation, voids may coalesce into a single larger hollow core. It is interesting that close-packed lattice (α-Ti) contains small holes in its structure, which account for 26% of the total crystal volume. In this case, there are octahedral and tetrahedral holes as illustrated in Fig. 3(c). We believe that the phase transformation/retransformation dynamic releases even more vacancies and increases Ti ions mobility.

Without electrical current, the Kirkendall effect may occur on Ti/TiO_2_ interface leading to the formation of vacancies, but the voids are much smaller than the microscale of the pores due to poor vacancy mobility and, consequently, the tubular morphology is not observed, as shown in Fig. 1(a–c). In a metallic conductor, the applied current would concentrate more on the surface of the microwire creating an electric field as illustrated in Fig. 3(c). The onset of a structural phase transition creates a huge amount of vacancies due to diffusion of ions in order to form another phase with a body-centered cubic crystal structure. The alteration of the atomic arrangement accompanied by dislocation of planes and bond shortening can induce strain localization between the low and high temperature crystal phase achieving movement and agglomeration of vacancies. The application of electrical current would lead to a high degree of mobility, delocalizing the vacancies, pulling them to the center, and forming huge voids. After the formation of voids, the flow of Ti ions would be through surface and lattice diffusion. In this case, we believe that the electrical current would also accelerate the diffusion process in order to consume the entire metallic core in a very narrow temperature interval and time. Extensive studies on the influence of electric field during oxidation were performed by Fromhold^36^,^37^. More recently, a self-consistent and quantitative understanding of the electric field role in the growing oxide film during oxidation process is presented^38^. However, the colossal diffusion phenomenon shown here has never been observed and not taken into account on these studies. It is well accepted that there is an electrostatic potential drop across the oxide layer, but quantitative understanding of the role of electric field remains incipient.

Summarizing, the study presented here reveal two major results in condensed matter physics. First, a colossal mass transfer physical phenomenon where the scenario combines structural phase transition, electrical current, and oxidation process at high temperatures. Second, as a consequence of mass transfer, a new route for hollow materials is anticipated. We believe that this new, simple, fast, and reproducible synthesis method to produce TiO_2_ microtubes can be extended to other metals showing structural phase transition at high temperature. We have repeated the above experiments using Cu, Ni, and Fe, exploring them under diverse temperature and current conditions. With the sole exception of Fe, which shows a structural phase transition at 912 °C from gamma to alpha phase, in no other case were we able to reproduce the results obtained here for Ti. The result reveals the importance of phase transition in the process. We consider that microtubes of other metals showing structural phase transition at high temperature can also be obtained. We believe that this study opens a novel avenue for both fundamental physics related to solid state diffusion and material science where design of hollow materials with potential smart functionalities can be obtained. So far, an envisaged analytical expression for the flux of ions involving electrical current which cause colossal mass transfer is not available and it is beyond the scope of this work. We hope that these results attract the attention of scientist interested in elucidating the microscopic mechanism leading to this colossal ions diffusion effect.

## Methods

TiO_2_ microtubes were fabricated by using titanium metal microwires with diameter of 127 μm in a horizontal quartz tube furnace. The high purity (99.99%) precursor Ti metallic microwire has been purchased from Alfa Aesar (temper as drawn). The oxidation process was carried out in air at temperatures up to T = 1050 °C by using 5 °C/min. The thermal oxidation process was accompanied by *in situ* electrical resistivity measurements using a four-point method. The electrical contacts are made of small drops of silver epoxy, which is cured at 160 °C. The microwire is perpendicularly placed on four parallel platinum wires, which are fixed on a platform made of alumina. In other words, the microwire is suspended on top of the four probes, not resting in any kind of surface. The structural properties of the as-synthesized microtubes and *in situ* measurements (as a function of temperature) were studied by using X-ray diffraction (XRD) performed in transmission geometry on a STADI-P diffractometer, from Stoe^®^ (Darmstadt, Germany), using MoKα_1_ radiation (λ = 0.7093 Å). Morphological properties were studied by scanning electron microscopy (JEOL FEG-SEM JSM 6701F). The Raman measurements were performed by using a triple spectrometer (T64000, HORIBA Jobin-Yvon) with a thermoelectric cooled CCD detector (Synapse, HORIBA Jobin-Yvon) equipped with a microanalysis option. The 532 nm line of an optically pumped semiconductor laser (Verdi G5, Coherent) was used as the excitation source. The laser power at the sample was maintained below 2 mW on a spot with a diameter of 2 μm (50 X Olympus objective). The measurements were performed in a near-backscattering configuration.

## Additional Information

**How to cite this article**: Rivaldo-Gómez, C. M. *et al*. New route for hollow materials. *Sci. Rep.* **6**, 32107; doi: 10.1038/srep32107 (2016).

## Supplementary Material

Supplementary Information

## Figures and Tables

**Figure 1:**
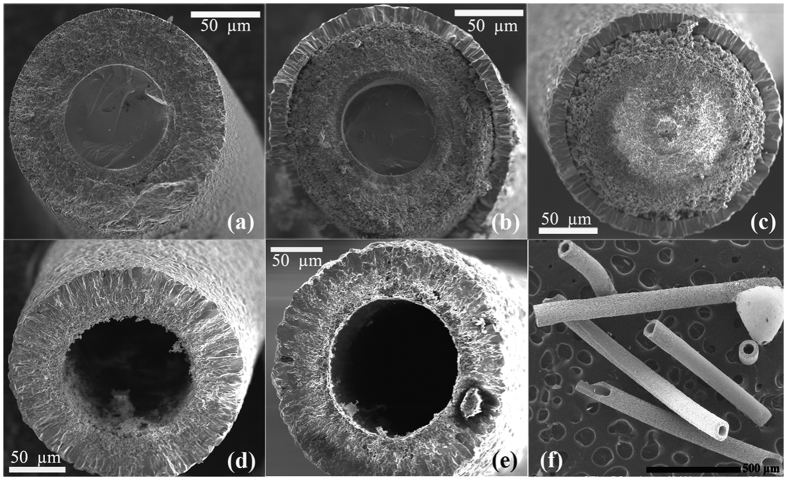
Core/shell wire turning into microtube. (Top panel) Scanning electron microscopy images obtained for three microwires subjected to a heat treatment up to T = 1050 °C for *t* = 0 (**a**), 1 h (**b**), and 2 h (**c**) without the passage of an electrical current. (Bottom panel) Transverse section images of microtubes obtained by thermal oxidation up to T = 1050 °C (*t* = 0 h) with an applied electrical current of 10 mA (**d**) and 0.1 mA (**e**). (**f**) Image of several segments of TiO_2_ microtubes.

**Figure 2:**
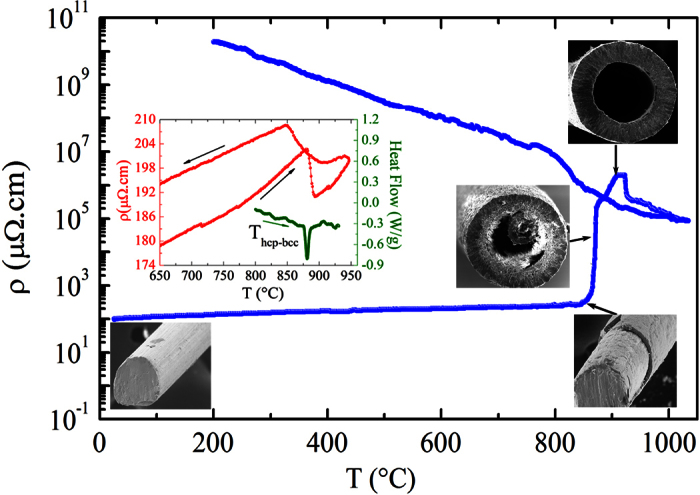
From microwire to microtube during oxidation process along with electrical current. Temperature dependence of the electrical resistivity obtained during the thermal oxidation of a metallic Ti microwire in air with an electrical current of 10 mA. The inset shows electrical resistivity (red curve) along with a DSC (green curve) curve of a metallic Ti microwire in argon atmosphere revealing, at T = 891 °C, a structural phase transformation from a hexagonal close-packed to a body-centered cubic crystal structure. SEM images illustrate different stages of the process.

**Figure 3:**
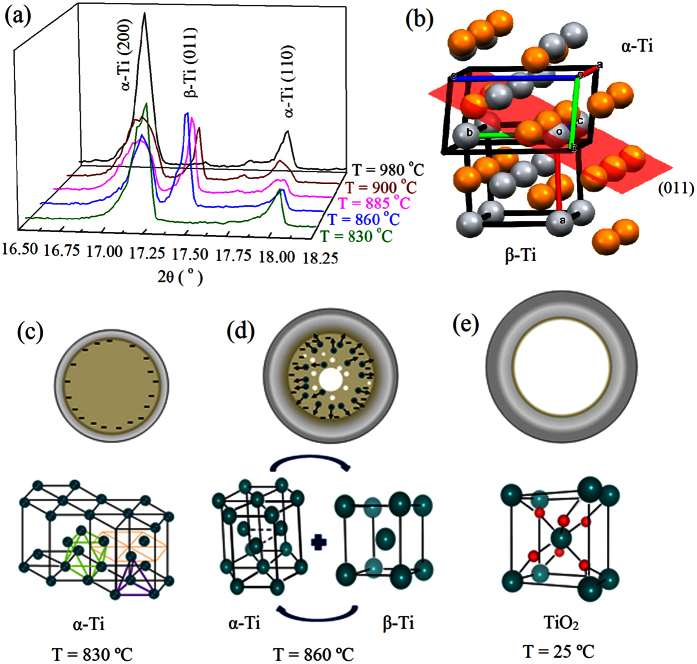
Crystal structure and morphology evolution. (**a**) *In situ* X-ray diffraction during the phase transformation showing the appearance and disappearance of a cubic β-Ti phase. (**b**) α- to β-Ti phase transformation displaying the movement of atoms along the (011) plane (orange atoms: α-Ti phase; grey atoms: β-Ti phase). The unit cells were rotated in order to provide a better visualization of the atoms. (**c–e**) Sketches of three main stages of the microtube formation process along with the crystal structure. At T = 830 °C, the electrical current travels on the surface of the wire – the lines represent the electric field in the metallic α-Ti phase. The structure shows two types of holes - octahedral holes with two staggered triangular planes of atoms and tetrahedral holes, which are formed by a planar triangle of atoms. (**d**) At T = 860 °C, the phase transition from α-Ti to β-Ti releasing a large number of Ti ions and vacancies. At room temperature, after the colossal mass transportation, a TiO_2_ microtube is observed.
