# Supplementary material for: New route for hollow materials
Source: Sci Rep. 2016 Aug 24;6:32107. doi: 10.1038/srep32107 (PMC4995455; doi:10.1038/srep32107)
Supplement: Supplementary Information [file srep32107-s1.doc]

**New route for hollow materials**

C. M. R. Gómez, F. F. Ferreira, G. Landi and J. A. Souza*

Centro de Ciências Naturais e Humanas, Universidade Federal do ABC, Santo André – SP, 09210-580, Brazil


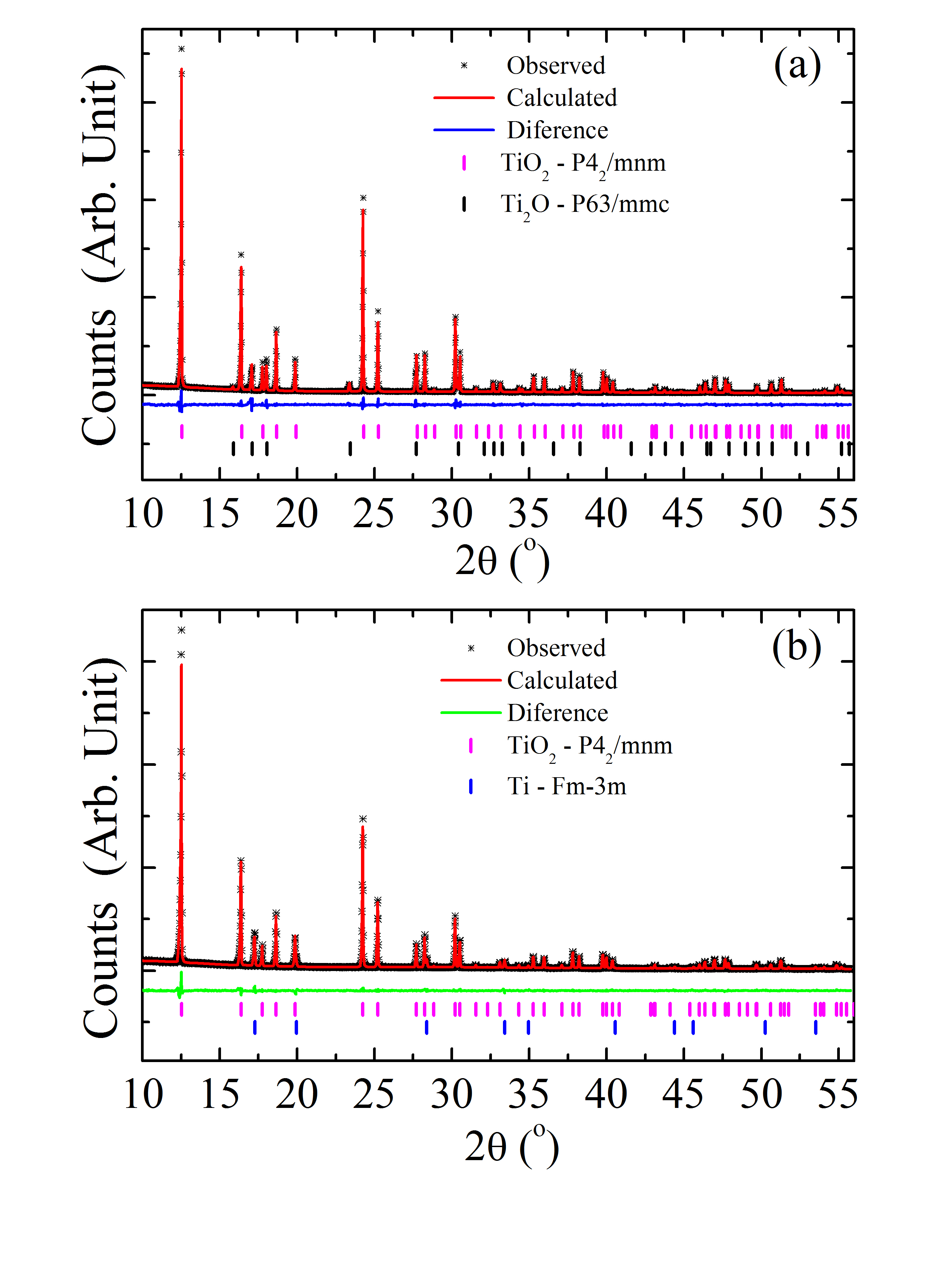


Figure 1S. X-ray diffraction patterns along with Rietveld refinements for Ti microwire oxidized up T = 1050 °C without (a) and with (b) the passage of electrical current of 10 mA. (a) The first phase, TiO_2_ rutile structure with *P4_2_/mnm* space group symmetry; the obtained lattice parameters are *a* = *b* = 4.5859(1) Å and c = 2.9553(1) Å and statistical parameters: *R_wp_* = 9.70%, *RBragg_TiO2* = 1.85%, *RBragg_Ti2O* = 7.88% and χ^2^ = 1.57, while the second phase corresponding to Ti_2_O has lattice parameters *a* = *b* = 2.9638(1) Å and *c* = 4.7700(3) Å and statistical parameters: *R_wp_* = 7.53%, *RBragg_TiO2* = 1.55%, *RBragg_Ti* = 4.60% and χ^2^ = 1.13, with *P6_3_/mmc* space group symmetry.


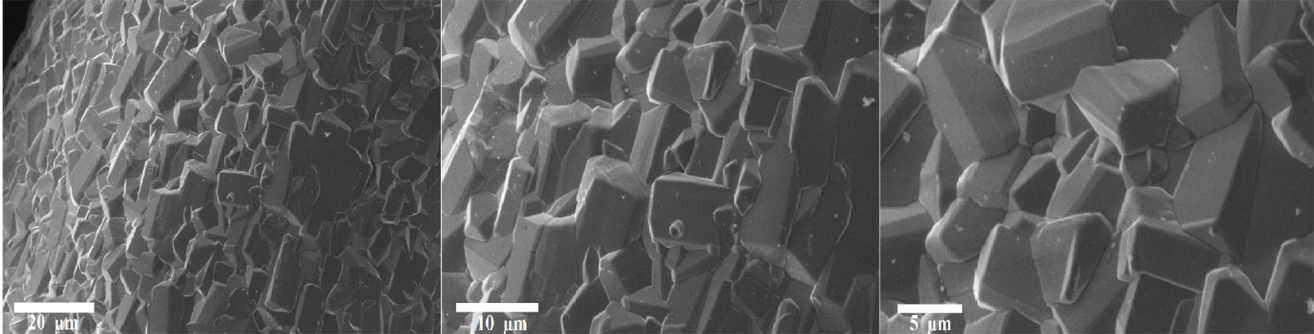


Figure 2S. (a) SEM images with different magnifications of the as obtained tube showing the surface microstructure.

*
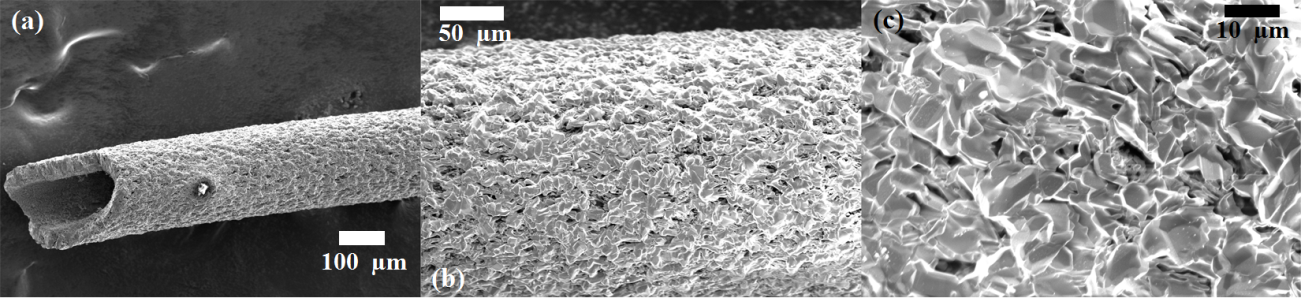
*

Figure 3S. SEM images of the annealed tube.

*
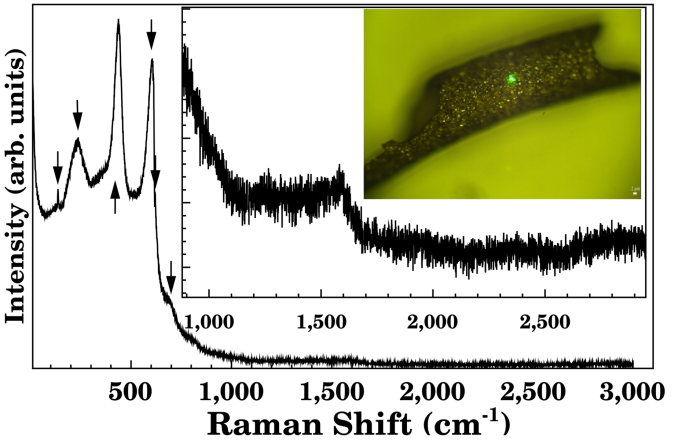
*

Figure 4S. Unpolarized Raman spectrum of TiO_2_ microtube. Arrows indicates assigned bands following Table 2S. The inset shows the spectral features between 800 – 2,800 cm^-1^ and a representative photograph of the focused laser on a microtube.

Table 1S. Raman bands assignment of the experimentally observed spectra of as-grown microtubes.

| ** (cm^-1^)** | **Band assignment** |
| --- | --- |
| 140 | E_g_(1) (anatase) |
| 232 | 2th order rutile |
| 441 | E_g_(rutile) |
| 606 | A_1g_(rutile) |
| 618 | E_g_(3) (anatase) |
| 694 | 2th order rutile |
| 826 | B_2g_ (rutile) |


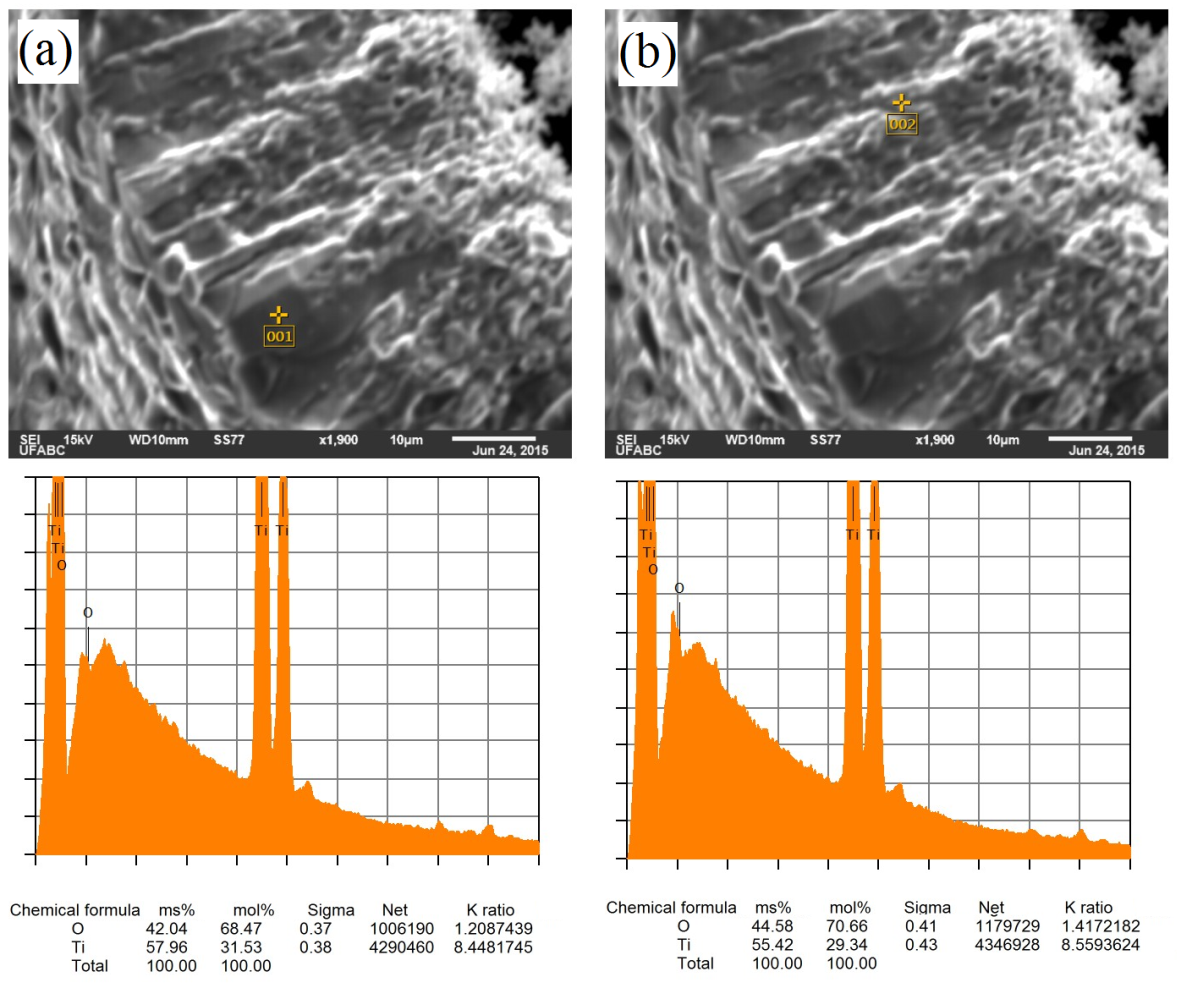


Figure 5S. Energy-dispersive spectroscopy (EDS) spectra at two different regions of the cross section of the synthesized TiO2 microtube along with tables of results. The EDS results confirm the TiO2 composition of the sample.


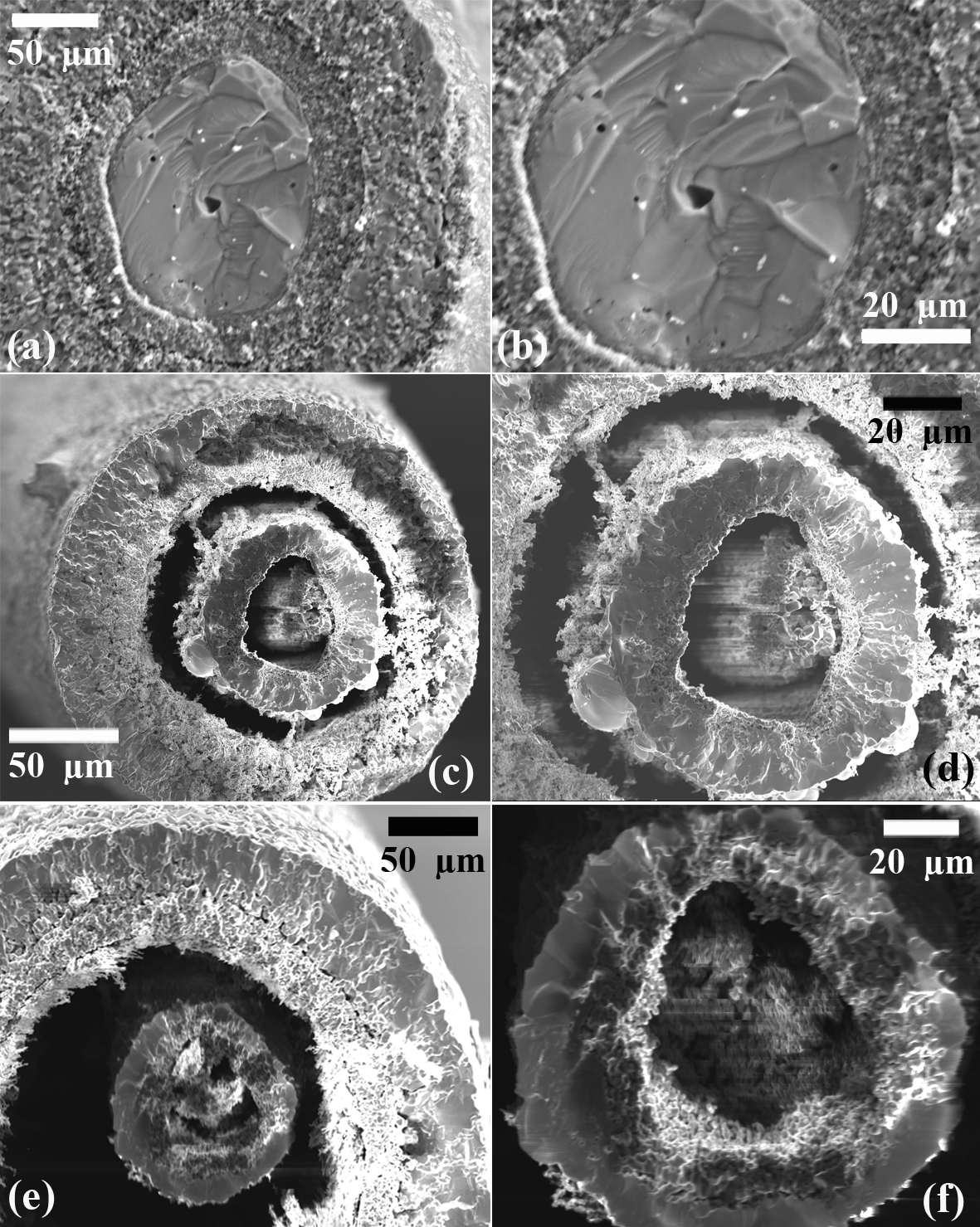


Figure 6S. Scanning electron microscopy images obtained for three microwires subjected to a heat treatment up to T ~ 840-850 ºC and T ~ 880-890 ºC and then aborted followed by cooling down the system to room temperature.


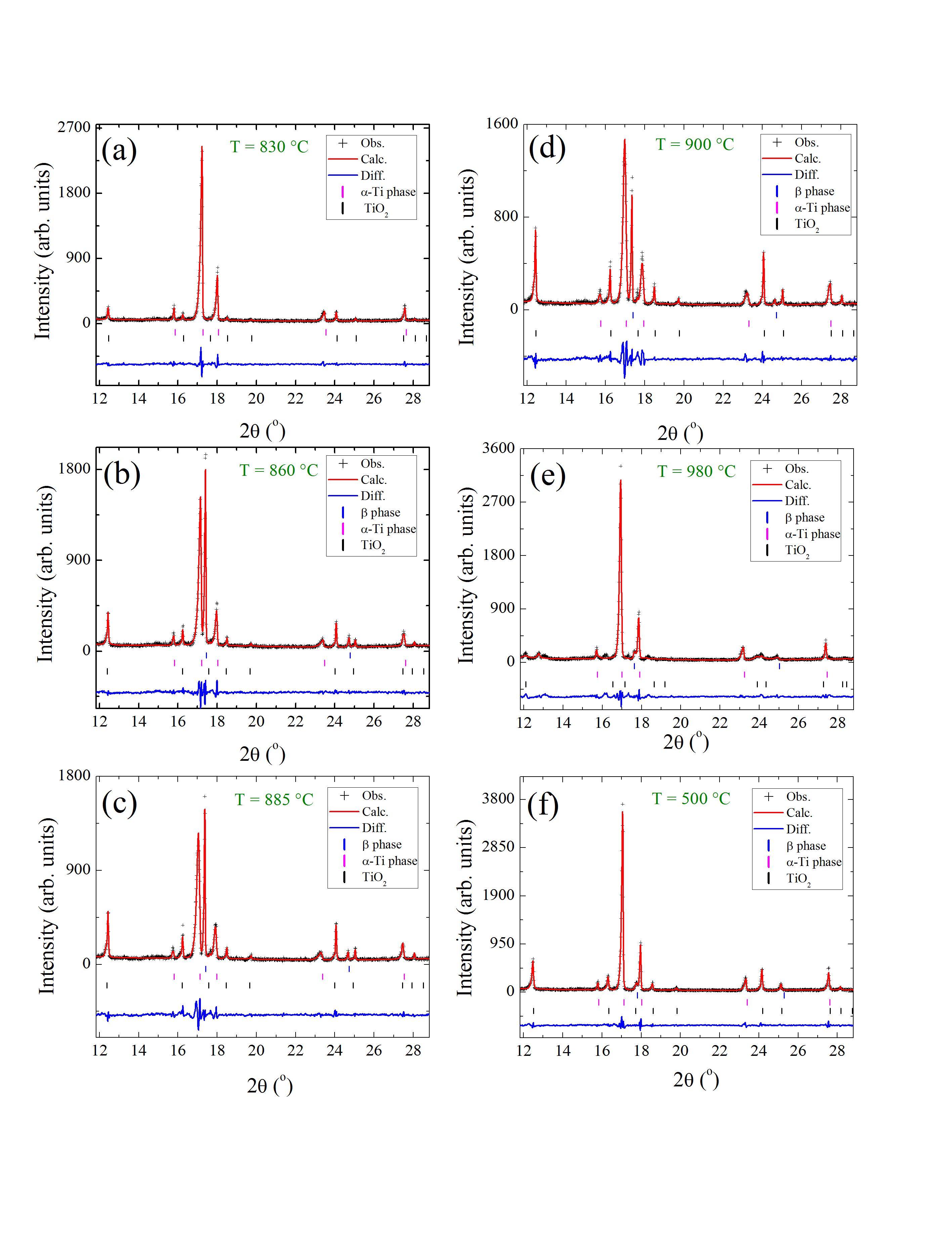


Figure 7S. X-ray diffraction patterns along with Rietveld refinements for Ti microwire as a function of temperature showing the evolution of the crystal phases. The statistical parameters were: (a) *R_wp_* = 11.05%, *R_Bragg_* = 1.00% and χ^2^ = 1.26; (b) *R_wp_* = 12.48%, *R_Bragg_* = 1.35% and χ^2^ = 1.45; (c) *R_wp_* = 12.10%, *R_Bragg_* = 1.21% and χ^2^ = 1.38; (d) *R_wp_* = 12.80%, *R_Bragg_* = 1.90% and χ^2^ = 1.46; (e) *R_wp_* = 15.10%, *R_Bragg_* = 3.03% and χ^2^ = 1.69; (f) *R_wp_* = 13.97%, *R_Bragg_* = 2.74% and χ^2^ = 1.61.

Table 2S. Volume fraction of the crystal phase as a function of temperature during the structural phase transition.

| Temperature (ºC) | α-Ti (wt%) | β-Ti (wt%) | TiO_2_ (wt%) |
| --- | --- | --- | --- |
| 30 | 100 | - | - |
| 830 | 75.9 | 4.0 | 20.1 |
| 860 | 48.9 | 23.2 | 27.9 |
| 885 | 53.4 | 12.5 | 34.1 |
| 900 | 53.7 | 7.4 | 38.9 |
| 930 | 59.3 | 2.4 | 38.3 |
| 980 | 51.9 | - | 48.1 |
